# Intravenous iron sucrose vs. blood transfusion in the management of moderate postpartum iron deficiency anemia: A non-randomized quasi-experimental study

**DOI:** 10.1016/j.heliyon.2022.e08980

**Published:** 2022-02-17

**Authors:** Rehana Arjuman Hye, Nur Sayeeda, G.M.Raihanul Islam, Jannatul Farjana Mitu, Mir Susmita Zaman

**Affiliations:** a Department of Obstetrics and Gynecology, Universal Medical College Hospital, Dhaka, Bangladesh; b Department of Obstetrics and Gynecology, Dhaka Medical College Hospital, Dhaka, Bangladesh; c Pi Research and Consultancy Center, Dhaka, Bangladesh; d Dhaka Medical College Hospital, India

**Keywords:** Iron sucrose, Blood transfusion, Postpartum anemia, Hemoglobin, Serum ferritin

## Abstract

**Introduction:**

Postpartum anemia is often over-treated with blood transfusion without clear indication despite having a potential alternative of parenteral iron therapy. The present study aimed to compare the efficacy of intravenous (IV) iron sucrose with blood transfusion in increasing the hematological parameters in postpartum women with moderate anemia.

**Methods:**

This prospective non-randomized quasi-experimental study was conducted among 44 hemodynamically stable postpartum women with moderate anemia (Hb 7–8 g/dl) in the Obstetrics department of Dhaka Medical College Hospital (DMCH) from January to June 2021. Among them, 22 patients received 600 mg of IV iron sucrose after 48 h of delivery for three subsequent days and the other 22 patients received two units of blood transfusion after 48 h of delivery in two subsequent days. The primary endpoint was increase in Hemoglobin (Hb) and serum ferritin level after 6 weeks of the intervention. Two-way repeated measures ANOVA (mixed factor ANOVA) was applied to compare between before and after effect in the two intervention groups.

**Results:**

Baseline Hb and ferritin were 7.4 g/dl and 73.5 μg/l in IV iron group and 7.3 g/dl and 73.2 μg/l in blood transfusion group. Mean Hb level was increased 4.2 g/dl in IV iron sucrose group and 4.5 g/dl in blood transfusion group at sixth week. Besides, serum ferritin level was increased 40.5 μg/l and 44.8 μg/l after six weeks in IV iron sucrose group and blood transfusion group respectively. Other hematological parameters like reticulocyte count, MCV, MCH, and MCHC also increased significantly after intervention in both groups. However, no significant difference was noticed in the change of hematological parameters in between the groups.

**Conclusions:**

The IV iron sucrose is as effective as blood transfusion in replenishing the hemoglobin and iron storage status in hemodynamically stable women with moderate post-partum anemia. This could be an effective alternative of blood transfusion in treating these patients, especially in resource-poor settings.

## 1 Introduction

Postpartum anemia (anemia after the delivery of a child) is a common but often neglected public health issue throughout the world (Zhao et al., 2019; Milman, 2011a, b). Almost one-fifth of the 300 thousand maternal deaths occurring each year globally is contributed by the peripartum hemorrhage and anemia (Maternal Mortality, 2019). The prevalence of postpartum anemia varies in different income class countries. While the prevalence ranges from 8 to 16% in high income and developed countries, it ranges from 50 to 80% in developing countries, especially in low income and rural populations (Milman, 2011a, b).

Iron deficiency anemia (IDA) during pregnancy in combination with excessive blood losses at delivery is a major contributor of postpartum anemia (Milman, 2011a, b). In Bangladesh, more than one-third of pregnant women suffer from some degrees of anemia (Chowdhury et al., 2015). Moreover, 16 out of every 1000 pregnant women experience peripartum hemorrhage in resource-poor settings of developing countries (Ngwenya, 2016). Hence, anemia during postpartum period is a major concern in Bangladesh with an estimated prevalence of almost 56% (Rahman et al., 2021). Postpartum anemia impairs the quality of life, reduces cognitive abilities, emotional stability, and increases the risk of depression and constitutes a significant health problem in women of reproductive age (Milman, 2011a).

According to the Network for Advancement of Transfusion Alternatives (NATA) recommendation, in postpartum period a hemoglobin (Hb) concentration of <10 g/dL indicates clinically significant anemia (Api et al., 2015). Different treatment modalities are recommended according to the grade of anemia. Oral iron supplementation should be the first-line treatment for mild IDA. Intravenous (IV) Iron therapy can be considered for moderate IDA (Hb level 8–9.5 g/dl) while blood transfusion is recommended for very severe IDA (Hb level <6 g/dl) (Api et al., 2015; Milman, 2011b). However, transfusions have traditionally been used to manage severe anemia in the pueperium, though in the absence of clear transfusion indications many women without severe symptoms also receive blood transfusions which potentially increases the risk of septicemia, hematological reactions, delayed wound healing and thromboembolism, particularly if they receive multiple transfusions (Chua et al., 2017; Patient Blood Management Guidelines: Module 4 Critical Care | National Blood Authority, 2013). Blood products are also a scarce and costly resource (Patient Blood Management Guidelines: Module 4 Critical Care | National Blood Authority, n.d.). Recent institutional guidelines and trial data suggest blood transfusions are likely to be appropriate in patients with severe anemia but not necessarily if alternative therapies like IV iron are available or if the individual is clinically well compensated (Api et al., 2015; Milman, 2011a, b; Prick et al., 2010). It is already reported that IV iron therapy can reduce the requirement for allogenic blood transfusion and can restore hemoglobin levels by an average of 2.5 g/dl within a week post infusion with peak effects observed at 3–6 weeks (Giannoulis et al., 2009; Neogi et al., 2019a). The safety profiles of these preparations are also ensured for the treatment of IDA during pregnancy and the postpartum period (Api et al., 2015).

Despite multiple existing evidences on comparison between IV and oral iron preparations (Bhandal and Russell, 2006; Ehrenstein et al., 2006; Radhika et al., 2019; Sultan et al., 2019; Van Wyck et al., 2007), there is a substantial lack in comparative study on effectiveness of IV iron sucrose with blood transfusion. A study conducted among anemic postpartum women of Saudi Arabia reported similar rise in Hb level after six weeks of intervention in both IV iron sucrose (2.15 g/dl) and blood transfusion group (2.35 g/dl) though the mean rise in ferritin level was higher in IV iron sucrose group (220%) than blood transfusion group (150%) (Khamaiseh and Tahat, 2020). Another clinical trial was registered with an aim to determine if intravenous iron is non-inferior to blood transfusion in correcting Hb deficit and replenishing iron stores in women with acute post-partum anemia, though the result is not available yet (Chua et al., 2017).

The objective of the present study was to compare the efficacy of IV iron sucrose with blood transfusion in increasing the Hb and serum ferritin level as well as other hematological parameters.

## 2 Methods

### 2.1 Study design and settings

This was a prospective non-randomized quasi-experimental study conducted in the department of Gynecology and Obstetrics of Dhaka Medical College Hospital (DMCH), Dhaka, Bangladesh from January to June 2021. Prior commencement of the study ethical clearance was obtained from the ethical review committee (ERC) of Dhaka Medical College.

### 2.2 Participants

All the postpartum patients admitted to the selected department of DMCH with the diagnosis of moderate anemia (defined by Hb level within 7–8 g/dl) were the study population. Convenience sampling according to the inclusion and exclusion criteria was used to include patients. Inclusion criteria were: (i) aged between 18 and 45 years, (ii) delivered by normal vaginal delivery or Cesarean section within the previous 24 h, (iii) Hb level within 7–8 g/dl. Exclusion criteria were: (i) delivery done more than 24 h ago, (ii) Hb level below 7 g/dl or above 8 g/dl, (iii) known hypersensitivity to parenteral iron preparation, (iv) recent blood transfusion, (v) associated cardiovascular, renal, hepatic dysfunction, (vi) infections including malaria, hook worm infestation, schistosomiasis, (vii) hereditary defects like sickle cell anemia, thalassemia, G6PD deficiency, bone marrow insufficiency etc, (viii) history of any bleeding tendency or clotting disorder, (ix) patients with placenta previa, placental abruption, postpartum hemorrhage or preeclampsia, and (x) hemodynamically unstable patients.

A total of 44 postpartum moderately anemic women were included in this study. An informed written consent was obtained from each patient regarding intervention. Eligible participants were randomly divided into two groups (each group containing 22 patients) for the interventions. Randomization was done with randomly permuted blocks according to a randomization schedule generated by the website randomization.com (http://www.randomization.com).

### 2.3 Intervention

First group received 600 mg of intravenous iron sucrose after 48 h of delivery for three subsequent days. Total required dose of iron was administered as 200 mg as a single dose and repeated three times. Iron sucrose was diluted in 200 ml of 0.9 % sodium chloride and administered slowly to avoid any adverse reactions. Second group received two units of blood transfusion after 48 h of delivery in two subsequent days. Both groups were provided with oral ferrous sulphate 300 mg daily for 5 weeks, started after one week of iron infusion or blood transfusion. Patients were observed for 24 h after the intervention for any adverse event and if occurred, patients were managed with standard treatment protocol.

### 2.4 Primary and secondary endpoints

Increase in Hb and serum ferritin level after 6 weeks of the intervention was the primary endpoint of the study. Increase in other hematological parameters including reticulocyte count, mean corpuscular volume (MCV), mean corpuscular hemoglobin (MCH), and mean corpuscular hemoglobin concentration (MCHC) (g/dl) was the secondary endpoint.

### 2.5 Data collection

Baseline demographic data (age and gravida of the patients) and hematological parameters were collected within 48 h of delivery and repeated after 6 weeks of intervention. Hematological parameters (Hb level, reticulocyte count, MCV, MCH, and MCHC) were determined by complete blood count conducted using the Sysmex XE-2100 hematology analyzer (Sysmex Corp. Kobe, Japan).

### 2.6 Statistical analysis

All statistical analyses were carried out using the STATA version 16.0. Descriptive statistics summarized and determined the sample demographic and hematological profile of patients. Continuous data were reported with mean and standard deviation (SD) and categorical data were summarized using frequencies and percentages. Associations between two or more qualitative variables were examined and assessed using Pearson Chi-square test. Efficacy measures included change in Hb, ferritin, and other hematological parameters from the baseline and were calculated using paired sample t-test. Two-way repeated measures ANOVA (mixed factor ANOVA) was applied to compare between before and after effect in the two intervention groups. A two-sided P value < 0.05 was considered to be statistically significant.

## 3 Results

A total of 44 postpartum women were enrolled in two different intervention groups (22 in IV iron sucrose group and 22 in blood transfusion group). Their average age as well as age distribution was similar (mean 23.7, SD 5.0 years of IV iron sucrose group and mean 24.7, SD 5.8 years of blood transfusion group). Baseline hematological parameters were also similar (Table 1).

**Table 1:** Baseline characteristics of the patients (n = 44).

Characteristics	IV Iron Sucrose	Blood transfusion	p-value
Age, mean (SD) (years)	23.70 (5.00)	24.70 (5.80)	0.37

Age category (years)			

≤20	9 (41.0)	6 (28.0)	0.24
21–30	11 (50.0)	12 (54.0)	
31–40	2 (9.0)	2 (9.0)	
>40	0 (0.0)	2 (9.0)	
Gravida			
Primigravida	6 (27.0)	7 (32.0)	0.07
Multigravida	16 (73.0)	15 (68.0)	

Hematological Parameters			

Hemoglobin (g/dl)	7.40 (0.32)	7.30 (0.31)	0.38
Serum ferritin (μg/l)	73.50 (3.00)	73.20 (3.00)	0.38
Reticulocyte count (%)	1.50 (0.60)	1.60 (0.80)	0.32
Mean corpuscular volume (MCV) (fl)	67.24 (5.00)	69.20 (3.00)	0.21
Mean corpuscular hemoglobin (MCH) (pg)	22.30 (2.60)	23.20 (3.60)	0.33
Mean corpuscular hemoglobin concentration (MCHC) (g/dl)	26.50 (1.90)	26.70 (1.20)	0.35

**Figure 1:**
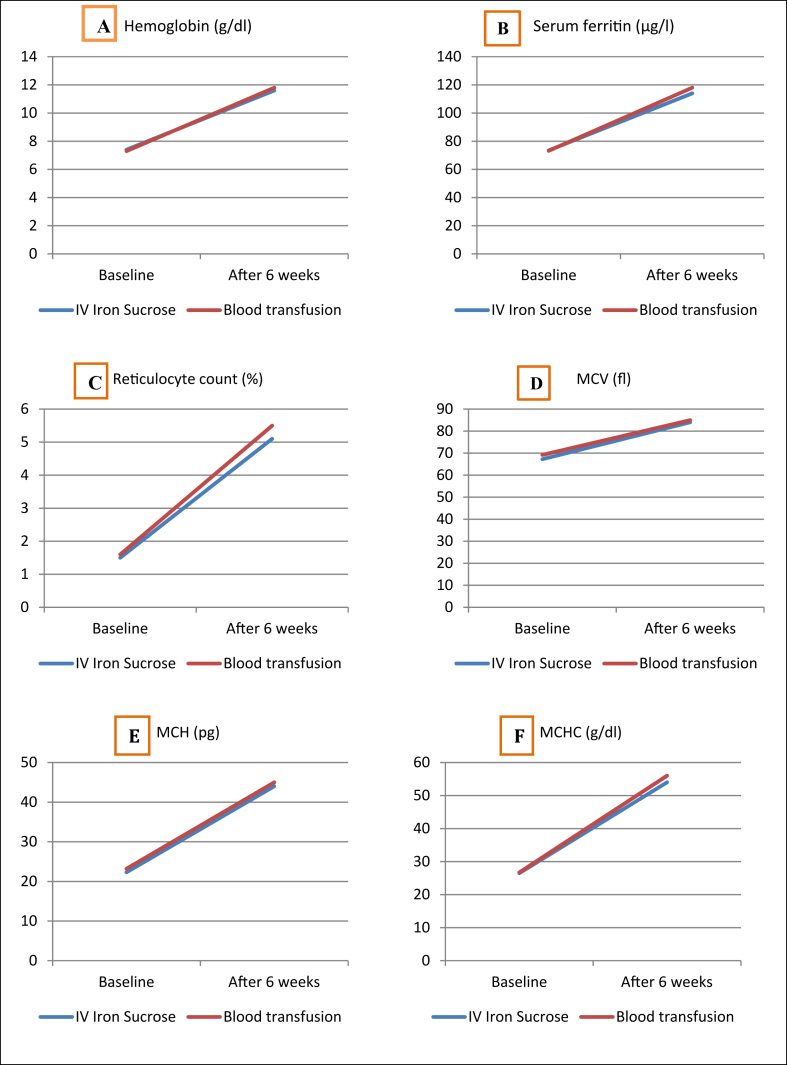
Change in hematological parameters (A: hemoglobin, B: serum ferritin, C: reticulocyte count, D: MCV, E: MCH, F: MCHC) before and after 6 weeks of intervention in IV iron sucrose and blood transfusion group (p-values were determined by the two-way repeated measures mixed factor ANOVA.).

All the hematological parameters increased significantly after intervention in both groups. Mean Hb level was increased 4.2 g/dl (56% from baseline) in IV iron sucrose group and 4.5 g/dl (61% from baseline in blood transfusion group. Mean serum ferritin level was increased 40.5 μg/l (55% from baseline) in IV iron sucrose group and 44.8 μg/l (61% from baseline) in blood transfusion group (Table 2). However, the change of different hematological parameter after intervention was identical in both intervention groups (Figure 1).

**Table 2:** Hematological parameters before and after treatment of the patients (n = 44).

Hematological parameters	IV Iron Sucrose			Blood transfusion		
	Baseline, n = 22	After 6 weeks, n = 22	p-value	Baseline, n = 22	After 6 weeks, n = 22	p-value
Hemoglobin (g/dl)	7.4 (0.32)	11.6 (1.0)	0.001	7.3 (0.31)	11.8 (1.2)	0.001
Serum ferritin (μg/l)	73.5 (3.0)	114 (2.5)	0.001	73.2 (3.0)	118 (3.6)	0.001
Reticulocyte count (%)	1.50 (0.60)	5.1 (1.3)	0.001	1.60 (0.80)	5.5 (1.8)	0.001
MCV (fl)	67.24 (5.0)	84 (1.4)	0.001	69.20 (3.0)	85 (2.3)	0.001
MCH (pg)	22.3 (2.6)	44 (3.2)	0.001	23.2 (3.6)	45 (3.1)	0.001
MCHC (g/dl)	26.5 (1.9)	54 (1.5)	0.001	26.7 (1.2)	56 (2.5)	0.001

## 4 Discussion

A large portion of women suffer from anemia during their postpartum period which increases the risk of short and long-term adverse health outcomes. A prompt and adequate management of postpartum anemia is necessary. Different treatment modalities are recommended by the guidelines according to the severity of anemia. For example, the Swiss Society for Gynecology and Obstetrics recommended that mild IDA (Hb 9.5–12 g/dl) should be treated with oral iron supplementation and moderate (Hb 8–9.5 g/dl) to severe (Hb < 8 g/dl) IDA should be treated with IV iron. Subcutaneous erythropoietin could also be considered in case of severe anemia (Breymann et al., 2011). On the other hand, the Network for Advancement of Transfusion Alternatives (NATA) recommended to consider erythropoietin along with IV iron in case of moderate to severe IDA (Hb 8–9.5 g/dl) and blood transfusion in case of very severe IDA (Hb < 6 g/dl) (Api et al., 2015).

Though blood transfusion is only recommended for very severe anemia, it was reported that many postpartum women are being transfused without any clear indication (Chua et al., 2017). Globally, almost 3% women receive blood products after their delivery (Thurn et al., 2019). A report from a tertiary hospital of Bangladesh stated that almost 10% of the postpartum women were treated with blood transfusion and anemia in pregnancy and postpartum period was the major cause of transfusion (Renuka et al., 2019). This high rate of transfusion potentially increases the risk of transfusion reactions, septicemia, delayed wound healing and thromboembolism, particularly in case of multiple transfusions (Chua et al., 2017). Moreover, the risk of transfusion reaction is almost double in postpartum women compared to non-pregnant women (Thurn et al., 2019).

The existing evidence argues the efficacy of parenteral iron compared to the oral iron in the management of mild to moderate anemia and this is faster and more effective at restoring hemoglobin and total body iron deficits compared to oral iron, and has the added advantage of avoiding gastrointestinal side effects which often interrupt the treatment compliance in the postnatal women (Bhandal and Russell, 2006; Ehrenstein et al., 2006; Radhika et al., 2019; Sultan et al., 2019; Van Wyck et al., 2007). However, due to excellent safety and tolerance profile as well as easy availability at a cheaper price IV iron (iron sucrose) could be an appropriate alternative to blood transfusions in hemodynamically stable moderate to severely anemic patients. We explored the efficacy of IV iron sucrose compared to blood transfusion in increasing the Hb and serum ferritin level as well as other hematological parameters in moderately anemic postpartum women.

Our result showed that all the hematological parameters increased significantly after intervention in both groups without any substantial difference. Mean Hb level was increased 4.2 g/dl in IV iron sucrose group and 4.5 g/dl in blood transfusion group at sixth week. Besides, serum ferritin level was increased 40.5 μg/l and 44.8 μg/l after six weeks in IV iron sucrose group and blood transfusion group respectively in our study. A study among postpartum women of Saudi Arabia receiving similar treatment regimen reported that mean Hb level increased 2.15 g/dl and 2.35 g/dl and serum ferritin level increased 15.2 μg/l and 10.5 μg/l in IV iron sucrose group and transfusion group respectively after a week of intervention (Khamaiseh and Tahat, 2020). Iron sucrose preparation showed high safety level in treatment of postpartum anemia without any interference in lactation (Christian et al., 2007; Neogi et al., 2019a, b; Pfenniger et al., 2012). Moreover, it reported a better efficacy in replenishing the Hb level and iron storage compared to other parenteral preparation like IV iron dextran (Samsudin et al., 2020) and intramuscular iron sorbitol (Wali et al., 2002).

Our study has several limitations. First of all, it was a single center study with a non-randomized, non-blinded allocation. So, the potential selection bias could not be rolled out. Moreover, the sample of the study represent only a subset of those who are moderately anemic by who definition (7–9.9 g/dl of hemoglobin), so the findings might not be generalized for overall patient population. Though, usual package labeling suggests five doses of IV iron sucrose, we used three infusions according to our hospital protocol. However, some recent trials suggest that a single dose (1g) of infused IV iron carboxymaltose is superior to multiple doses of IV iron sucrose, which should also be considered. Besides, ferumoxytol and iron derisomaltose, both of which are single infusion formulations might an alternative to iron sucrose and iron carboxymaltose (Adkinson et al., 2018; Pollock and Muduma, 2021; Rognoni et al., 2016). Another major limitation of the present study was the clinical outcomes in the mother or secondarily in the offspring was not considered. Moreover, patients were discharged with advice of oral iron therapy, tracing the patients’ compliance was not possible.

## 5 Conclusions

Our study evidenced that intravenous iron sucrose could be an effective alternative to blood transfusion in the management of hemodynamically stable women with moderate post-partum anemia. This will be useful for patients with clinical or other contraindication of blood transfusion and in under-resourced settings.

## Declarations

### Author contribution statement

Rehana Arjuman Hye, G. M. Raihanul Islam and Susmita Zaman: Conceived and designed the experiments; Performed the experiments; Analyzed and interpreted the data; Contributed reagents, materials, analysis tools or data; Wrote the paper.

Nur Sayeeda: Conceived and designed the experiments; Analyzed and interpreted the data; Contributed reagents, materials, analysis tools or data; Wrote the paper.

Jannatul Farjana Mitu: Conceived and designed the experiments; Performed the experiments; Contributed reagents, materials, analysis tools or data; Wrote the paper.

### Funding statement

This research did not receive any specific grant from funding agencies in the public, commercial, or not-for-profit sectors.

### Data availability statement

Data will be made available on request.

### Declaration of interests statement

The authors declare no conflict of interest.

### Additional information

No additional information is available for this paper.
